# Whole genome sequencing reveals the independent clonal origin of multifocal ileal neuroendocrine tumors

**DOI:** 10.1186/s13073-022-01083-1

**Published:** 2022-08-03

**Authors:** Netta Mäkinen, Meng Zhou, Zhouwei Zhang, Yosuke Kasai, Elizabeth Perez, Grace E. Kim, Chrissie Thirlwell, Eric Nakakura, Matthew Meyerson

**Affiliations:** 1 Department of Medical Oncology, Dana-Farber Cancer Institute, 450 Brookline Avenue, Boston, MA 02215 USA; 2 Cancer Program, Broad Institute of Harvard and MIT, Cambridge, MA USA; 3 Department of Surgery, University of California, San Francisco, CA USA; 4 Department of Pathology, University of California, San Francisco, CA USA; 5 Research Department of Oncology, UCL Cancer Institute, London, UK; 6 School of Medicine and Health, University of Exeter, RILD Building, Exeter, UK; 7 Departments of Genetics and Medicine, Harvard Medical School, Boston, MA USA

**Keywords:** Small bowel, Small intestinal neuroendocrine tumors, Multifocality, Whole genome sequencing, Independent clonal origin

## Abstract

**Background:**

Small intestinal neuroendocrine tumors (SI-NETs) are the most common neoplasms of the small bowel. The majority of tumors are located in the distal ileum with a high incidence of multiple synchronous primary tumors. Even though up to 50% of SI-NET patients are diagnosed with multifocal disease, the mechanisms underlying multiple synchronous lesions remain elusive.

**Methods:**

We performed whole genome sequencing of 75 de-identified synchronous primary tumors, 15 metastases, and corresponding normal samples from 13 patients with multifocal ileal NETs to identify recurrent somatic genomic alterations, frequently affected signaling pathways, and shared mutation signatures among multifocal SI-NETs. Additionally, we carried out chromosome mapping of the most recurrent copy-number alterations identified to determine which parental allele had been affected in each tumor and assessed the clonal relationships of the tumors within each patient.

**Results:**

Absence of shared somatic variation between the synchronous primary tumors within each patient was observed, indicating that these tumors develop independently. Although recurrent copy-number alterations were identified, additional chromosome mapping revealed that tumors from the same patient can gain or lose different parental alleles. In addition to the previously reported *CDKN1B* loss-of-function mutations, we observed potential loss-of-function gene alterations in *TNRC6B*, a candidate tumor suppressor gene in a small subset of ileal NETs. Furthermore, we show that multiple metastases in the same patient can originate from either one or several primary tumors.

**Conclusions:**

Our study demonstrates major genomic diversity among multifocal ileal NETs, highlighting the need to identify and remove all primary tumors, which have the potential to metastasize, and the need for optimized targeted treatments.

**Supplementary Information:**

The online version contains supplementary material available at 10.1186/s13073-022-01083-1.

## Background

Small intestinal neuroendocrine tumors (SI-NETs) are the most common neoplasms of the small bowel with an estimated annual age-adjusted incidence ranging from 0.67 to 1.12 per 100,000 persons [1–3]. SI-NETs originate from enterochromaffin cells of the digestive tract, and most tumors arise in the terminal ileum [4, 5]. SI-NETs are usually well-differentiated tumors characterized by a low proliferation rate, but also by a high percentage of distant metastases at diagnosis [6]. The 5-year survival rate is less than 50% in patients with metastatic disease [1, 6]. The only curative treatment of SI-NETs is complete surgical resection. Development of targeted therapies has been impeded by the lack of apparent driver genes in SI-NETs.

Previous high-throughput sequencing studies, which have primarily focused on targeted gene panel and whole exome sequencing, have reported low somatic mutation rates in SI-NETs [7–11]. The most frequent genomic alteration identified to date is loss of heterozygosity (LOH) at chromosome (chr) 18, occurring in 70% of tumors [12–14]. Other recurrent whole chromosome and whole chromosome arm copy-number alterations (CNAs) have been observed in 10–30% of SI-NETs, including gains of chromosomes 4, 5, 7, 14, and 20 [7–10]. The only recurrent mutations identified in SI-NETs are loss-of-function mutations in cyclin-dependent kinase inhibitor 1B (*CDKN1B*) in approximately 8–10% of tumors [8, 15]. Furthermore, a recent whole genome sequencing (WGS) study of 2520 metastatic solid tumors reported SI-NETs to rarely harbor a candidate driver mutation or germline predisposition variant. A total of 34 samples in the study had no identified drivers, and 18 of these samples were SI-NETs [16].

The majority of large-scale sequencing studies to date have concentrated on sequencing single primary tumors from each patient. Multiple synchronous lesions, however, have been observed in up to 50% of SI-NET patients [17, 18]. The molecular mechanisms underlying these multifocal lesions are not yet understood. Recently, two high-throughput sequencing studies of multifocal SI-NETs by us and others have suggested these tumors to develop independently [19, 20]. Our high-throughput sequencing-based copy-number profiling of 40 multifocal ileal NETs revealed distinct patterns of chr18 allelic loss in individual tumors from the same patient, suggesting these tumors originate independently and that no specific germline allele on chr18 is targeted by somatic LOH [19]. Additionally, Elias et al. performed WGS of 61 tumor samples (42 primary tumors and 19 metastases) from 11 patients with multifocal SI-NET to study the evolutionary trajectory of multifocal SI-NETs within single patients [20]. They observed lack of shared somatic variation among the primary tumors within the patients, supporting the independent clonal origin of multifocal SI-NETs.

To obtain a comprehensive molecular genomic characterization of multifocal SI-NETs and to confirm previous findings, we performed WGS of 90 tumor samples (75 primary tumors and 15 metastases) from 13 patients with multifocal ileal NETs. Our analysis of somatic single-nucleotide variants (SNVs), insertion-deletion mutations (indels), CNAs, and structural variants (SVs) revealed substantial genomic diversity among the multifocal ileal NETs, indicating that these tumors develop independently. We also provide evidence showing that metastases in a multifocal ileal NET patient can occasionally originate from different primary tumors.

## Methods

### Samples

Each patient provided informed consent in accordance with the protocols approved by the Institutional Review Boards of the Dana-Farber Cancer Institute and the University of California San Francisco. The initial sample material consisted of 87 de-identified synchronous primary tumors, 20 metastases and matched adjacent normal ileal mucosa and/or whole blood specimens from 13 multifocal ileal NET patients, who underwent surgery at the University of California San Francisco. The clinical characteristics of the patients are summarized in Table 1. All patients had been diagnosed with well-differentiated ileal NETs (grades 1–2) [21], which was confirmed post-operatively, and developed metastatic disease. The tissue specimens were snap-frozen in liquid nitrogen after surgical excision and stored at − 80 °C. Tumor purity was assessed for each specimen using hematoxylin and eosin (H&E) staining before sequencing. Samples with tumor purity ≥ 20% were selected for DNA extraction, resulting in a total of 78 synchronous primary tumors and 16 metastases.

**Table 1:** Patient cohort characteristics

**Patient**	**Gender**	**Average age [range]**	**Average number of primary tumors [range]**	**T**^**a**^	**N**^**a**^	**M**^**a**^	**Stage**^**a**^	**Grade**^b^
744	Male	64 [53–75]	7 [2–18]	T3	N1	M1a	IV	G2
760	Male			T2	N0	M1a	IV	G2
772	Male			T3	N2	M1a	IV	G1
825	Male			T4	N1	M1a	IV	G1
848	Female			T3	N1	M1a	IV	G1
850	Male			T3	N2	M0	III	G2
852	Male			T3	N2	M1c	IV	G1
876	Female			T3	N2	M0	III	G1
947	Female			T4	N2	M1b	IV	G1
952	Female			T3	N1	M0	III	G1
1060	Male			T3	N2	M1a	IV	G2
1076	Male			T4	N2	M1c	IV	G1
1089	Male			T2	N2	M0	III	G1

^a^TNM staging was based on American Joint Committee on Cancer 8th edition

^b^Grade was based on WHO Classification 5th edition

### DNA extraction

Genomic DNA was extracted from the whole blood specimens using the QIAamp® DNA Blood kit (Qiagen, Germantown, MD) according to the manufacturer’s instructions, whereas gDNA extraction of fresh-frozen primary ileal NETs, metastases, and normal ileal mucosa specimens was performed at the Genomics Platform (GP), Broad Institute of MIT, and Harvard, Cambridge, MA. For three primary tumors, the amount of extracted DNA was too low to proceed with WGS.

### Whole genome sequencing

A total of 75 synchronous primary tumors, 16 metastases, and 18 normal tissue specimens entered WGS. Library construction, WGS, and preprocessing of raw sequencing reads were carried out at the GP [22]. Briefly, gDNA libraries were sequenced on HiSeq X Ten (Illumina, San Diego, CA) to generate 151-bp paired-end reads with a mean target depth of 60 × coverage for tumor specimens, and 30 × coverage for the normal tissue specimens. Sequenced reads were aligned to GRCh38/hg38 reference assembly using BWA–MEM [23] and duplicate-marked with Picard tools [24]. GATK was utilized for base score recalibration and local indel re-alignments [25]. One metastasis specimen failed quality control of WGS data and was excluded from the study. The mean coverage achieved was 80.5 × [60.0–171.3 ×] for tumor specimens and 75.3 × [30.5–102.3 ×] for normal tissue samples (Additional file 1: Table S1).

### Somatic variant calling

SNVs and indels were called with Mutect2 [26] from GATK v4.1.9.0 [27] and Strelka2 v2.9.10 [28] and functionally annotated with GATK’s Funcotator using default parameters. Normal ileal mucosa samples were used as matched normal tissue, apart from patient 952, from which only whole blood sample was available. Additionally, a panel of sequences derived from normal samples was applied for Mutect2, including both normal ileal mucosa and whole blood specimens from ileal NET patients. Further filtering for SNVs and indels included removal of variants with coverage ≤ 6 reads and population variants with minor allele frequency (MAF) > 0.001 (gnomAD genome data). All the coding variants were individually visualized with Integrative Genomics Viewer (IGV) v2.7.2 to exclude those present only in the same direction reads or repetitive genomic regions as likely artifacts. Only noncoding variants called by both Mutect2 and Strelka2 were included in the study. CNAs were called using GATK’s somatic copy-number variant discovery workflow. Non-overlapping genomic intervals were used for collecting read counts. CNAs with median logR-ratio > 0.15 and <  − 0.15 were included in the study with an aim to capture major CNA events while filtering out noisy signals. It is possible that using these thresholds some low-abundance subclonal events may have been filtered out. CNA breakpoints located in chromosomal centromeres or within 1 Mb at the end of a chromosome, and CNAs < 10 kb in size were excluded from the study. SVs were called using SvABA v1.1.3 with default parameters [29]. SVs < 10 kb in size, defined by the distance of two breakpoints if they were located at the same chromosome, and with breakpoints locating in a centromeric region were disregarded. All SVs were individually visualized with IGV to exclude likely artifacts.

### Mutation significance analysis

MutSig2CV [30] was used to identify genes that were mutated more often in the tumors than expected by chance given the inferred background mutation processes. The analysis was performed for somatic coding region SNVs and indels using both patient- and tumor-level information. Due to the low somatic mutation rate in SI-NETs, we increased the sample size for this analysis by combining our data with previously published high-throughput sequencing data of SI-NETs [8, 16], resulting in 176 tumors from 99 SI-NET patients. For the patient-level analysis, all samples from the same patient were collapsed together and the analysis included a union of unique mutations from each patient. For the tumor-level analysis, tumors with highly similar somatic coding region SNV and indel profiles were removed by MutSig2CV from the analysis. Default parameters were applied, and FDR-adjusted *P* < 0.1 was considered statistically significant.

### Clustering analysis

We used fishhook [31] to identify statistical enrichment or depletion of SNVs and indels in the genomes of all primary ileal NETs and metastases. The Gamma-Poisson regression model was corrected by incorporating covariates of genomic features. The covariates used included common fragile sites from HGNC BioMart [32], known retrotransposons annotated by RepeatMasker [33], and nucleotide context, including GC, CpG, and TpC contents. A non-overlapping bin size of 10 kb was used for SNVs and 100 kb for indels. False discovery rate (FDR; Benjamini-Hochberg) < 0.1 was considered statistically significant.

### Signaling pathway analysis

Signaling pathway analysis was performed for all 90 tumor samples. We used a statistical test (hypergeometric distribution) to determine whether certain Reactome pathways were enriched among the mutated genes in the primary ileal NETs and metastases [34]. Synonymous variation was excluded from the analysis. Overall, 560 out of 880 genes were found in Reactome v75, where 1378 pathways were hit by at least one of them. The probability score for each pathway was FDR corrected using the Benjamini–Hochberg method. FDR-adjusted *P* < 0.1 was considered statistically significant. As described in the results, no pathways were statistically significant after FDR correction.

### Mutation signature analysis

Mutation signature analysis was performed for all 90 tumor samples. Mutation signature analysis focused on single base substitutions (SBS) in 96 trinucleotide contexts. Mutational matrices were created using SigProfilerMatrixGenerator [35] with default parameters. SigProfilerExtractor [36] was used to perform de novo extraction of a maximum of five mutational signatures per sample and decomposition of the signatures into COSMIC mutational signatures v3.2 [37].

### Chromosome mapping

Germline single-nucleotide polymorphisms (SNPs) were called with HaplotypeCaller [38] from GATK v4.1.9.0 using allele-specific filtering workflow with a truth sensitivity filter level of 99%. Chromosome mapping was performed for all recurrent CNAs that were present in multiple tumors from at least two ileal NET patients as described previously [19]. First, heterozygous germline SNPs were identified from the normal tissue samples using the following filters: read depth > 10 and variant allele frequency between 0.4 and 0.6. The allelic depths of these SNPs were retrieved from the corresponding tumor samples if the total read depth of a given SNP was > 10 in the tumor samples. Next, a binomial test was applied to the read counts of the reference and alternative alleles of each SNP with the null hypothesis of 0.5, meaning that both alleles were expected to occur in half of the reads. FDR correction was performed for all SNPs across the segment in question. SNPs with FDR-adjusted *P* < 0.05 were considered as informative SNPs. For tumors with < 1000 shared informative SNPs, FDR-adjusted *P* < 0.1 was applied. The deleted and amplified chromosome alleles were assigned for each informative SNP by comparing the read counts of reference and alternative alleles. We acknowledge that this approach may not be optimal for capturing subclonal CNAs or allelic imbalance in samples with low tumor purity.

## Results

### Patient cohort

Patient cohort characteristics are detailed in Table 1. Most were white males (8/13, 62%). The median age at surgery was 64 years (range 53–75), and all patients had developed metastatic disease. The number of synchronous primary tumors varied from two to 18 among the multifocal ileal NET patients. The total number of metastases per patient was unknown. WGS was successfully performed for 75/91 (82%) primary ileal NETs and 15 metastases, including nine lymph node and six liver metastases (Additional file 1: Table S1).

### Mutational landscape of primary ileal NETs and metastases

**Fig. 1:**
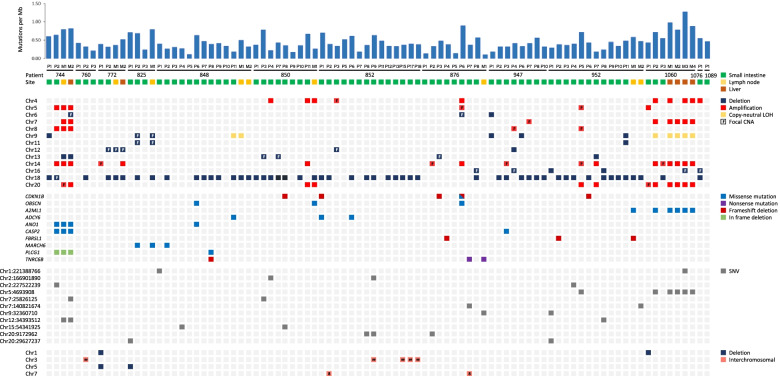
Mutational analysis of 75 synchronous primary ileal NETs and 15 metastases. Top panel, somatic mutation rate per Mb for all 90 tumors. Tumor sites are indicated by colored boxes. P, primary tumor; M, metastasis. Second panel, the most recurrent somatic copy-number alterations (CNAs) identified in both primary ileal NETs and metastases that are present in at least two ileal NET patients. The CNAs are arranged by chromosome number order. Focal CNAs are marked with “F” to differentiate them from whole chromosome and whole chromosome arm events. Third panel, the ten most frequently mutated genes in primary ileal NETs and metastases present in two or more ileal NET patients. Fourth panel, the most recurrent somatic noncoding variants identified in primary ileal NETs and metastases that are present in more than one ileal NET patient. Bottom panel, all recurrent structural variants (SVs) that are present in at least two ileal NET patients. The SVs are arranged by chromosome number order. Chromosome number of the second breakpoint of the interchromosomal rearrangement has been marked in the colored box

WGS data analysis identified 124,550 somatic SNVs and indels across all 90 sequenced tumor samples, consisting of 1447 coding region variants and 123,103 noncoding region variants (Additional file 1: Table S1). The average somatic mutation burden was 0.41 mutations per megabase (mut/Mb) per primary ileal NET (range 0.11–0.89) and 0.63 mut/Mb per metastasis (range 0.10–1.27) (Fig. 1, Additional file 1: Table S1). The mean number of coding variants per sample was 15 in primary ileal NETs (range 2–71) and 20 in metastases (range 7– 35), whereas the mean number of noncoding variants per sample was 1256 (range 346–2736) and 1926 (range 288–3914), respectively (Additional file 1: Table S1). Most of the coding variants were either missense (65%) or silent mutations (27%) (Additional file 2: Fig. S1, Additional file 3: Table S2).

Copy-number analysis identified 107 regions of somatic deletion, 101 regions of somatic amplification and nine copy-neutral LOH events across all 90 tumor samples (Additional file 1: Tables S1 and S3). The majority of the CNAs were either whole chromosome or chromosome arm events (58%) and present in both primary ileal NETs and metastases (Additional file 2: Fig. S2). The mean number of CNAs per sample was 2.15 in primary ileal NETs (range 0–15) and 3.73 in metastases (0–10) (Additional file 1: Table S1). We also identified 147 intrachromosomal (47%) and 168 interchromosomal somatic SVs (53%) across all 90 tumor samples (Additional file 1: Tables S1 and S4). Intrachromosomal SVs included deletions (33%), duplications (31%), translocations (24%), and inversions (13%). The mean number of SVs per sample was 3.68 in primary ileal NETs (range 0–23) and 2.60 in metastases (0–12) (Additional file 1: Table S1). The higher mean value in primary ileal NETs can be explained by a few individual tumors that harbored markedly more SVs than the rest of the tumors.

### CNAs are the most recurrent somatic alterations in multifocal ileal NET patients

**Fig. 2:**
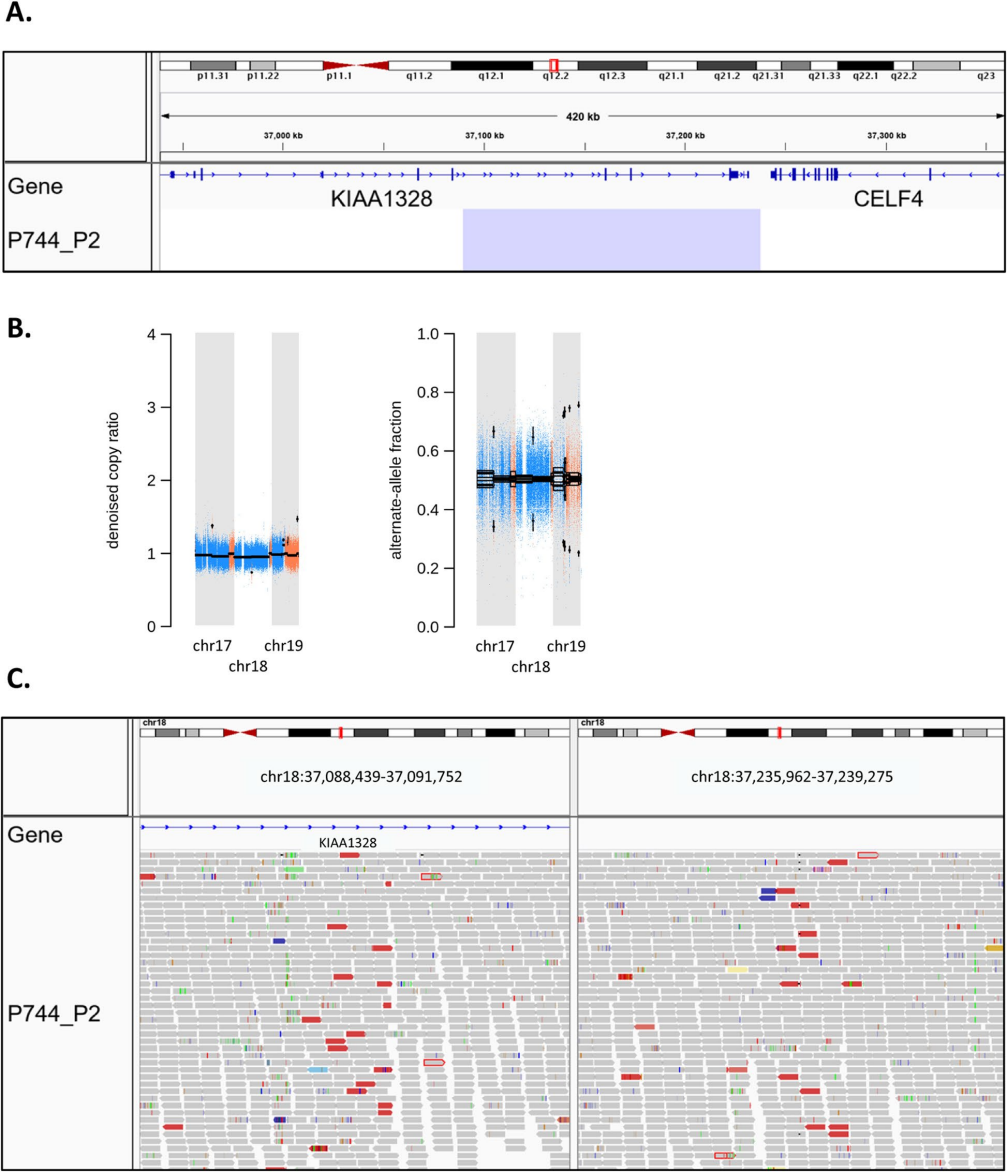
A minimally targeted region on chr18q12.2.** A** Integrative Genomics Viewer (IGV) view of a 148 kb deletion at chr18q12.2 in a primary ileal NET. The deletion overlaps with a part of a gene, *KIAA1328*, and a part of an intergenic region between *KIAA1328* and the adjacent gene, *CELF4*. **B** The copy ratio and allele fraction segments. **C** IGV view of the sequencing reads at the breakpoints of the deletion. The reads are colored by insert size. Reads that are colored red have larger than expected inferred sizes, indicating a deletion

The most recurrent somatic genomic alterations in primary ileal NETs and metastases were whole chromosome and chromosome arm events. Chr18 LOH (51/90, 57%) was clearly the most frequent CNA followed by amplifications of chr14 (12/90, 13%), 20 (10/90, 11%), 4 (9/90, 10%), and 7 (7/90, 8%) (Fig. 1, Additional file 1: Table S3). Additionally, six minimally targeted regions of size < 5 Mb were identified, 4p15.2, 10q11.21, 14q32.2–32, 18q12.2, 19p13.11, and 20p13-12.3, each present in tumors of at least two multifocal ileal NET patients (Additional file 2: Fig. S3). One of the regions, 18q12.2, contained only a part of a single gene, *KIAA1328*, resulting in loss of the last three exons of the gene, as well as a part of an intergenic region between *KIAA1328* and the adjacent gene, *CELF4*, which harbors a lncRNA (AC015961.1) and a candidate cis-regulatory element (cCRE) of CTCF-only group as reported by the ENCODE Encyclopedia [39] (Fig. 2). All the other regions were comprised of multiple genes, including a few known cancer genes according to COSMIC Cancer Gene Census v92: *SLC34A2* on 4p15.2, *RET* on 10q11.21, *HSP90AA1* on 14q32.31, and *JAK3* on 19p13.11. No additional coding region variants, however, were observed in these genes.

### TNRC6B is a candidate tumor suppressor gene in SI-NETs

We also identified 96 recurrently mutated genes harboring nonsynonymous mutations across all tumor samples (Additional file 2: Fig. S4, Additional file 3: Table S2). Many of the genes (66/96, 69%) were mutated in both primary ileal NETs and metastases, though, typically only in the tumors of one ileal NET patient at a time (Additional file 2: Fig. S2 and S4). Only two genes among all recurrently mutated genes, *CDKN1B* (5/90, 6%) and *OBSCN* (3/90, 3%), displayed mutations in the tumors of more than two ileal NET patients (Fig. 1). Furthermore, we identified only one recurrently mutated gene, *DROSHA*, that harbored somatic alterations affecting the same exact genomic location in tumors from more than one ileal NET patient. Two tumors from two different patients (P852_P15 and P952_P10) displayed different missense mutations in this location: c.1282G > A, p.D428N and c.1282G > C, p.D428H (Additional file 2: Fig. S4). This location is not a known mutation site in *DROSHA*. A clear majority of the somatic alterations in the recurrently mutated genes were missense mutations (85%) (Additional file 2: Fig. S4). We observed three genes that harbored frameshift deletions and/or nonsense mutations in tumors from multiple ileal NET patients: *CDKN1B*, *FBRSL1*, and *TNRC6B* (Fig. 1). Additional larger deletions affecting *CDKN1B* and *TNRC6B* were identified in five primary ileal NETs in both cases (Additional file 2: Fig. S5). *CDKN1B* was also affected by copy-neutral LOH in one primary ileal NET. Lastly, ten out of 96 (10%) recurrently mutated genes were known cancer genes according to COSMIC Cancer Gene Census v92: *ARID2*, *BCL11B*, *BCOR*, *CDKN1B*, *DROSHA*, *FAT4*, *HIP1*, *NUTM1*, *PLCG1*, and *ROBO2* (Additional file 2: Fig. S4). Nonsynonymous mutations were also identified in 51 additional cancer genes in single primary ileal NETs and metastases, including *ALK*, *DAXX*, *KRAS*, and *MEN1* (Additional file 2: Fig. S6).

To identify genes showing statistical evidence of positive selection for mutations in SI-NETs, we performed a mutation significance analysis for somatic coding region SNVs and indels. Due to the low somatic mutation rate in SI-NETs, we combined our data with previously published high-throughput sequencing data of SI-NETs [8, 16], resulting in 176 tumors from 99 SI-NET patients. The analysis was performed for both patient- and tumor-level data separately. *CDKN1B* was the most significant gene in both analyses (*Padj* = 6.3 × 10^−12^, patient-level data; *Padj* = 1.5 × 10^−11^, tumor-level data) (Additional file 1: Tables S5 and S6). The patient-based analysis identified one additional significant gene, *ZNF845* (*Padj* = 2.6 × 10^−2^), whereas the tumor-based analysis identified four, *TNRC6B* (*Padj* = 2.9 × 10^−4^), *ZNF780B* (*Padj* = 4.2 × 10^−2^), *RPP30* (*Padj* = 6.4 × 10^−2^), and *CASQ1* (*Padj* = 8.7 × 10^−2^). Only two significant genes, *CDKN1B* and *TNRC6B*, were mutated across the studies. A closer look at previous high-throughput sequencing data of SI-NETs revealed three somatic nonsense mutations and two frameshift deletions in *TNRC6B* among 195 sequenced sample pairs, as well as ten deletions of chr22 [7, 8, 16, 20]. Together with our data, *TNRC6B* is affected in approximately 8% of SI-NETs and metastases.

### The majority of recurrent noncoding region and structural variants are patient specific

In terms of the noncoding genome, recurrent noncoding variants affecting the same exact genomic location were observed in both primary ileal NETs and metastases, the latter harboring most of the variants (Fig. 1, Additional file 2: Fig. S2). As in the case of recurrently mutated genes, the majority of the recurrent noncoding variants (6505/6533, 99.6%) were present in the tumors of only one ileal NET patient at a time (Additional file 3: Table S2). Further visualization of the remaining 28 variants resulted in the identification of 18 somatic recurrent noncoding variants (0.3%) that were displayed concurrently in the tumors of more than one ileal NET patient (Additional file 1: Table S7). We also detected 27 recurrent SVs, four of which were present in tumors from more than one ileal NET patient (Fig. 1, Additional file 1: Table S4). Two of the recurrent SVs were deletions affecting genes *PEX14* and *CASZ1* on chr1 and *SGCD* on chr5, respectively. The breakpoints of the remaining two recurrent interchromosomal rearrangements located in introns of the following genes: *AC034195.1* on chr3, *WWOX* on chr16, *HOXA3* on chr7, and *SGCZ* on chr8.

### CDKN1B frameshift deletions cause a statistically significant cluster of indels on chr12

In the absence of apparent recurrent genomic driver alterations among the tumors of multifocal ileal NET patients, we next looked for potential enrichment of SNVs and indels in the genomes of primary ileal NETs and metastases. We identified one statistically significant cluster of SNVs on chr2 (*Padj* = 7.2 × 10^−2^) caused by a somatic intronic SNV in *FOXN2* (g.chr2:48340593 T > C), which was present in eight tumors from one ileal NET patient (Additional file 2: Fig. S7, Additional file 3: Table S2). Also, one statistically significant cluster of indels was observed on chr12 (*Padj* = 2.4 × 10^−2^), consisting of four *CDKN1B* frameshift deletions from three different ileal NET patients and one intronic deletion in *APOLD1* (Additional file 2: Fig. S7, Additional file 3: Table S2).

### No evidence for significantly mutated signaling pathways in multifocal ileal NET patients

Due to the lack of recurrently mutated genes among the multifocal ileal NET patients, we examined if the mutated genes fell into the same signaling pathways. We identified 13 pathways with nominally significant *P*-value (< 0.05); however, none of the pathways remained significant after multiple testing correction (Additional file 1: Table S8).

### Mutational signatures SBS1 and SBS5 occur in all primary ileal NETs and metastases

**Fig. 3:**
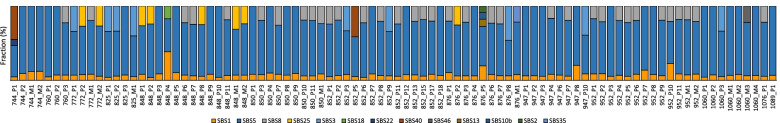
Mutational signatures of 75 synchronous primary ileal NETs and 15 metastases. The mutation signatures of each tumor sample have been decomposed into COSMIC mutational signatures v3.2. P, primary tumor; M, metastasis; SBS, single base substitution

The most common substitution types were transitions C > T and T > C that accounted for 30% and 24% of all the detected mutations, respectively. SBS signatures 1 and 5 were observed in all tumor samples and the only signatures present in 36% of the tumors, the latter contributing the most to the mutational profiles of primary ileal NETs and metastases (Fig. 3). Both signatures are considered clock-like signatures, indicating that the number of mutations correlates with the age of the individual. Additionally, SBS signatures 3 (10%), 8 (39%), and 25 (9%) were identified in multiple tumors from various ileal NET patients and the signatures were mutually exclusive. SBS8 and SBS25 are unknown signatures, whereas SBS3 has been associated with defective homologous recombination-based DNA damage repair. No clear differences were observed between the mutational signatures of primary ileal NETs and metastases. However, the patterns and fractions of the signatures varied between the tumors from the same patient.

### Synchronous primary tumors arise independently from the normal ileal mucosa

**Fig. 4:**
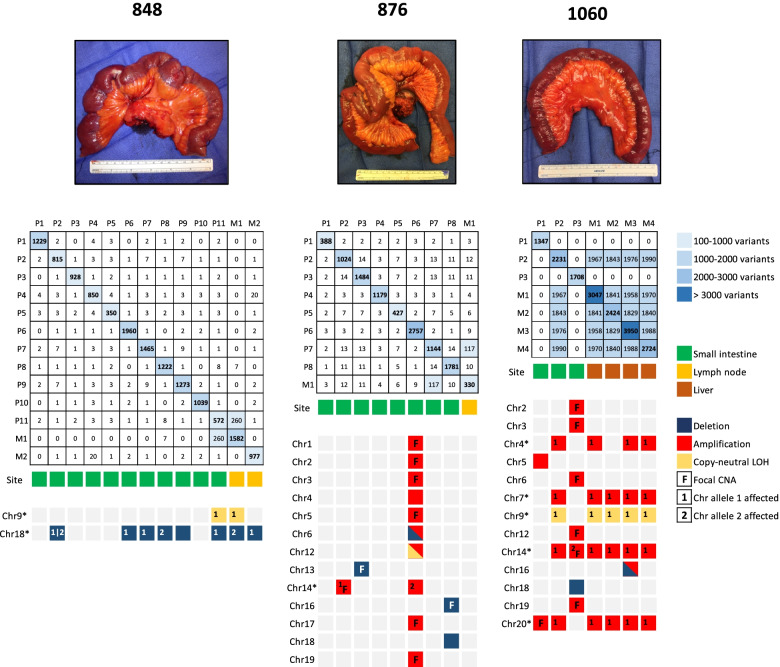
Somatic tumor evolution in three multifocal ileal NET patients. Upper panel represents the resected segments of ileum. Image from the patient 848 has been previously published under the terms of CC BY 4.0 as Fig. 1A in Zhang et al. [19]. Middle panel shows shared SNVs and indels between each tumor pair within a multifocal ileal NET patient. The somatic nature of the shared SNVs and indels was verified each time < 100 variants were shared between two tumors. Also, the presence of the shared variants in other tumors of the same patient was checked to avoid missing subclonal variants. Tumor sites are indicated by colored boxes. Bottom panel consists of somatic CNAs identified in each tumor. The CNAs are arranged in chromosome number order. Focal CNAs are marked with “F” to differentiate them from whole chromosome and whole chromosome arm events. Chromosome mapping was performed for chromosomes marked with an asterisk. P, primary tumor; M, metastasis

Next, we studied the clonal relationship of the tumors within each multifocal ileal NET patient. WGS data of multiple tumor samples were available from 11 patients. Pairwise comparison of the numbers of shared SNVs and indels indicated that multifocal primary tumors within each patient arise independently from the normal ileal mucosa (Fig. 4, Additional file 2: Fig. S8). On average, only 1.9 (0.08%) somatic SNVs and/or indels (range 0–14; 0–0.7%) were shared between the primary tumors. All shared variants, except one in *JADE2* (c.1465C > T, p.R489C) observed in two out of eight primary tumors in patient 947, were noncoding variants. WGS data of metastases were available from eight of the patients. Based on the numbers of shared SNVs and indels, we identified the putative primary tumors of origin for the sequenced metastatic tumors in seven patients as follows (Fig. 4, Additional file 2: Fig. S8): P744_P2 for P744_M1 and P744_M2, P772_P2 for P772_M1 and P772_M2, P825_P2 for P825_M1, P848_P11 for P848_M1, P876_P7 for P876_M1, P952_P2 for P952_M1 and P952_P10 for P952_M2, and P1060_P2 for P1060_M1-4. None of the primary tumors in patient 850 shared SNVs or indels with the metastasis (Additional file 2: Fig. S8). However, WGS data were not available from three of the patient’s primary tumors, suggesting that one of those tumors could be the origin for the metastasis. Intriguingly, two different dissemination patterns were identified in patients with multiple metastases. Metastases were either clonal, originating from a single primary tumor, or independent, originating from two separate primary tumors within a patient (Fig. 4, Additional file 2: Fig. S8). We did not observe any common somatic alterations or mutational signatures between the metastasized primary tumors that would separate them from the other primary tumors.

Although CNAs were the most recurrent somatic genomic alterations identified in primary ileal NETs and metastases, none of them were present in all tumors of the same patient. Additional chromosome mapping of the most frequent CNAs among the tumor samples revealed that primary tumors and metastases from the same patient can present different CNA patterns, including gains or losses of the same parental allele, a different parental allele consistent across the whole chromosomal arms, or different parental alleles in the short (p) and long (q) arms (Additional file 1: Table S9). Gains or losses of different parental alleles were identified in chromosomes 4, 14, and 20, as well as chromosomes 13 and 18 in the multifocal ileal NET patients, respectively (Fig. 4, Additional file 2: Fig. S8). The clear majority of the observed CNA patterns were concordant with the results from the pairwise comparisons of the amounts of shared SNVs and indels between the tumors within each patient. However, there were two occasions, one in patient 744 and the other in patient 848, where clonal tumors had gained or lost different parental alleles in a subset of CNAs (Fig. 4, Additional file 2: Fig. S8).

## Discussion

To better understand the molecular mechanisms underlying the growth and development of multifocal SI-NETs, we whole genome sequenced 75 primary tumors, 15 metastases, and the corresponding normal tissue samples from 13 multifocal ileal NET patients. Low somatic mutation rate was observed across all tumor samples, which is in line with the previous literature [7–11, 16]. CNAs were the most recurrent somatic alterations identified. Chr18 LOH was present in 57% of primary tumors, representing the most recurrent somatic alteration in multifocal ileal NETs, along with amplifications of chromosomes 4, 7, 14, and 20 in 9–13% of tumors. These results are consistent with previous high-throughput sequencing studies of both uni- and multifocal ileal NETs [7, 8, 10, 19, 20]. In addition to the previous literature, we observed copy-neutral LOH of chr9 in 8% of tumors. Interestingly, we identified a small deletion on 18q12.2 in one out of 44 primary ileal NETs displaying chr18q LOH. The deletion was supported by both the CNA and SV data, and it primarily affected a part of *KIAA1328*, but also an intergenic region between *KIAA1328* and the adjacent gene, *CELF4*. *KIAA1328* encodes a protein called hinderin, which has been shown to bind to SMC3, a subunit of the cohesin complex, which plays an important role in mediating sister chromatid cohesion, homologous recombination, and DNA looping [40]. Currently, there are no functional data available for the lncRNA (AC015961.1) or cCRE that are located in the intergenic region affected by the deletion. One of the previous high-throughput sequencing studies of SI-NETs has reported another small deletion on 18q12.2 in one out of 22 primary NETs displaying chr18q LOH in the region, as well as a deletion breakpoint at 18q12.2 in a second primary NET [7]. Both the deletion and the breakpoint affected *CELF4*, which encodes an RNA-binding protein (RBP) implicated in the regulation of pre-mRNA alternative splicing [41]. The protein is used as a part of RBP-based models to predict the survival of colorectal cancer (CRC) patients [42]. Also, a rare intronic germline variant in *CELF4* has been associated with CRC risk [43]. Neither *KIAA1328* nor *CELF4* have previously been implicated in small bowel cancer.

Along with the recurrent CNAs, we observed recurrent frameshift and/or nonsense mutations, as well as larger deletions affecting two genes, *CDKN1B* and *TNRC6B*, in 11% and 9% of tumors across multiple ileal NET patients, respectively. *CDKN1B* has previously been implicated as a potential tumor suppressor gene in SI-NETs, linking cell cycle dysregulation in their tumorigenesis [8, 15]. In addition to the previous studies, we demonstrated that *CDKN1B* frameshift deletions cause a statistically significant enrichment of indels on chr12 in multifocal ileal NETs, strengthening the role of *CDKN1B* as a driver gene in SI-NETs and suggesting that the gene displays a specific mutational pattern in the tumors. Like *CDKN1B*, *TNRC6B* was identified as one of the genes showing statistical evidence of positive selection for mutations in SI-NETs, suggesting a candidate tumor suppressor role for this gene in a subset of tumors. Trinucleotide repeat containing 6 (TNRC6) proteins, including TNRC6A, TNRC6B, and TNRC6C, are important for miRNA- and siRNA-mediated gene silencing through their functions within RNA-induced silencing complex [44]. Downregulation of TNRC6B has been suggested to contribute to tumorigenesis of different cancers, such as prostate cancer and lung adenocarcinoma [45, 46]. Inhibition of TNRC6B has been shown to lead to acceleration of cell proliferation and deceleration of cell adhesion in hepatoma cell lines [47]. We did not observe other apparent candidate driver genes among the multifocal ileal NET patients.

Further comparison of the tumors within each multifocal ileal NET patient confirmed that multifocal primary tumors arise independently from the normal ileal mucosa. The primary tumors rarely shared any SNVs, indels, or SVs, and the observed recurrent CNAs were not present in all tumors of the same patient. Additional chromosome mapping revealed that tumors from the same patient can display different CNA patterns, including gains or losses of either parental allele. In the majority of the cases, where the tumors had gained or lost a different parental allele, the change was consistent across the whole chromosomal arms. We observed, however, one patient with a tumor that had lost different parental alleles in the short (p) and long (q) arms of chr18. This case has been discussed in our previous paper, where we speculate that there are two different mechanisms that may lead to this event; either homologous recombination after which one of the recombined copies is lost during tumorigenesis, or two independent genomic events [19]. We also detected variation in the patterns and fractions of mutational signatures between tumors from the same patient. Intriguingly, we show that multiple metastases in the same patient can originate from one or several primary tumors. Our results are corroborated by recent findings of Elias et al. [20] and have an important clinical implication, supporting the concept that identification of all multifocal primary tumors by careful palpation of the entire jejunum and ileum is essential at the time of surgery [48]. We could not detect common somatic alterations among the metastasized primary tumors in our data.

Together with previous high-throughput sequencing studies on multifocal SI-NETs [19, 20], our data confirm the lack of shared driver genes among these tumors, suggesting that multifocal ileal NETs are not driven by genomic alterations that are detectable with our current genome sequencing and analysis approaches. Additionally, our previous discovery of distinct chr18 LOH patterns in multifocal ileal NETs from the same patient excludes the possibility of a germline loss-of-function mutation in a tumor suppressor gene on chr18 as a cause for SI-NETs [19]. These findings suggest that SI-NETs could be mainly driven by epigenetic mechanisms, or alternatively arise from morphologically normal small intestine as a result of a field cancerization. Also, the tumor microenvironment in the ileum may play a role in the growth and development of SI-NETs.

## Conclusions

Our study indicates notable genomic diversity among multifocal ileal NETs, suggesting that these tumors develop independently. We identified potential loss-of-function gene alterations in *TNRC6B*, a candidate tumor suppressor gene in a small subset of ileal NETs, as well as a minimally deleted region on chr18q12.2, providing new candidate genes to study to better understand the molecular mechanisms of SI-NETs. Additionally, we observed that multiple metastases in the same patient can originate from either one or several primary tumors, which highlights the need to identify and remove all primary tumors, which have the potential to metastasize. Altogether, our results suggest the tumorigenesis of SI-NETs is unlikely to be driven exclusively by genomic alterations and underscore the need of a deeper understanding of the molecular mechanisms that underlie SI-NETs and to apply that knowledge toward development of new and effective treatments.

## Supplementary Information

**Additional file 1:** **Table S1.** WGS sample information and somatic genomic alteration counts. **Table S3.** Filtered copy-number alteration calls. **Table S4.** Filtered structural variant calls. **Table S5.** MutSig2CV results for patient-level information. **Table S6.** MutSig2CV results for tumor-level information. **Table S7.** Recurrent non-coding variants. **Table S8.** Results of the signaling pathway analysis. **Table S9.** Results of chromosome mapping analysis

**Additional file 2: Figure S1.** Classification of coding variants in 75 primary ileal NETs and 15 metastases. **Figure S2.** Overlap of somatic variation between primary ileal NETs and metastases. **Figure S3.** Minimally targeted regions of size < 5Mb. **Figure S4.** Recurrently mutated genes in 75 primary ileal NETs and 15 metastases. **Figure S5.** Deletions affecting *CDKN1B* and *TNRC6B*. **Figure S6.** Known cancer genes mutated in single primary ileal NETs and metastases. **Figure S7.** Statistically enriched regions of SNVs and indels in 75 primary ileal NETs and 15 metastases. **Figure S8.** Somatic tumor evolution in multifocal ileal NET patients.

**Additional file 3:** **Table S2.** Filtered SNV and indel calls.

## Data Availability

The WGS data generated and analyzed in this study are accessible at the European Genome-phenome Archive (EGA) website under the accession number EGAS00001006294 (https://ega-archive.org/studies/EGAS00001006294) [22].

## Abbreviations

cCRE: Candidate cis-regulatory element
CDKN1B: Cyclin-dependent kinase inhibitor 1B
CNA: Copy-number alteration
FDR: False discovery rate
Indel: Insertion-deletion mutation
LOH: Loss of heterozygosity
SBS: Single base substitution
SI-NET: Small intestinal neuroendocrine tumor
SNP: Single-nucleotide polymorphism
SNV: Single-nucleotide variant
SV: Structural variant
TNRC6B: Trinucleotide repeat containing 6 B
WGS: Whole genome sequencing
