# Supplementary material for: Whole genome sequencing reveals the independent clonal origin of multifocal ileal neuroendocrine tumors
Source: Genome Med. 2022 Aug 3;14:82. doi: 10.1186/s13073-022-01083-1 (PMC9351068; doi:10.1186/s13073-022-01083-1)
Supplement: Supplementary file 2 — Additional file 2: Figure S1. Classification of coding variants in 75 primary ileal NETs and 15 metastases. Figure S2. Overlap of somatic variation between primary ileal NETs and metastases. Figure S3. Minimally targeted regions of size < 5Mb. Figure S4. Recurrently mutated genes in 75 primary ileal NETs and 15 metastases. Figure S5. Deletions affecting CDKN1B and TNRC6B. Figure S6. Known cancer genes mutated in single primary ileal NETs and metastases. Figure S7. Statistically enriched regions of SNVs and indels in 75 primary ileal NETs and 15 metastases. Figure S8. Somatic tumor evolution in multifocal ileal NET patients. [file 13073_2022_1083_MOESM2_ESM.pdf]

Supplemental Figures 1-8

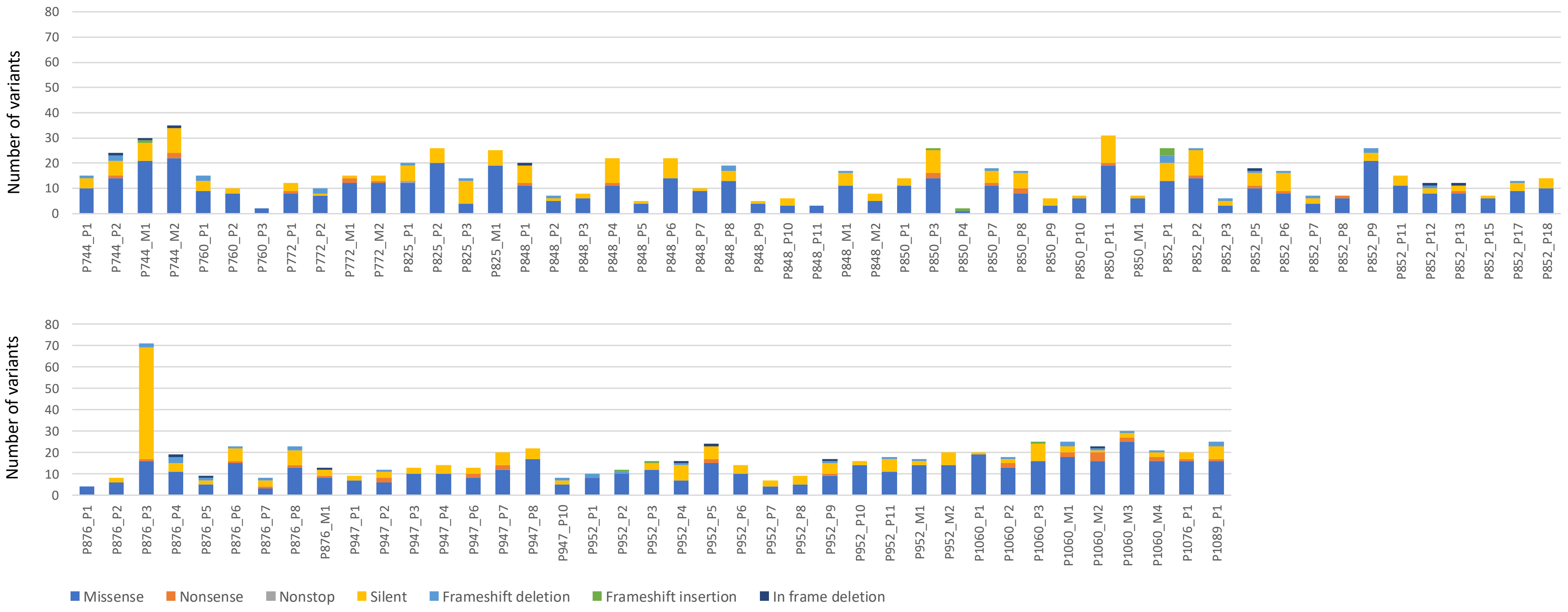

Figure S1. Classification of coding variants in 75 primary ileal NETs and 15 metastases. Majority of the coding variants were either missense (65%) or silent mutations (27%) followed by frameshift deletions (4%) and nonsense mutations (3%).

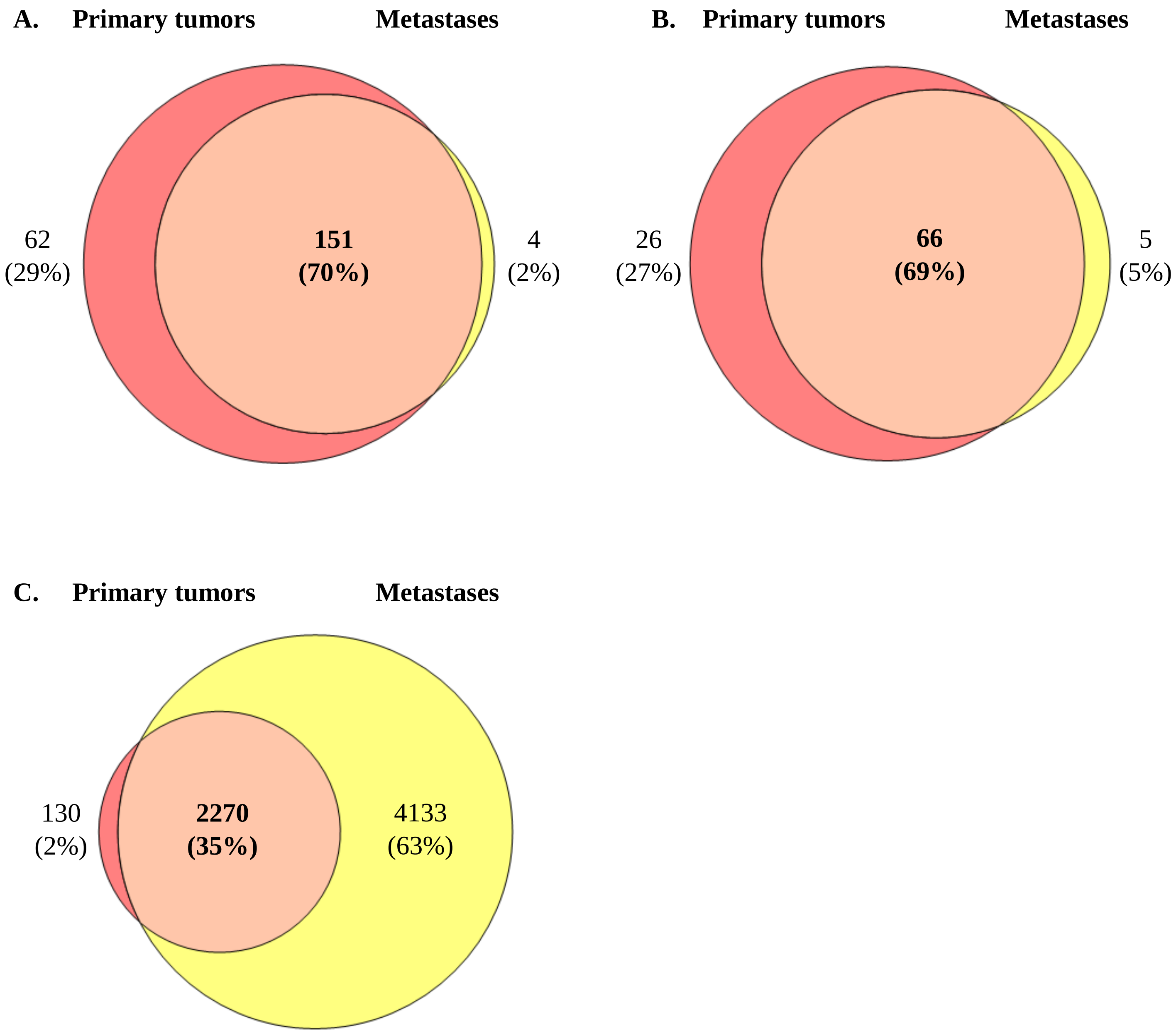

Figure S2. Overlap of somatic variation between primary ileal NETs and metastases. A) Majority of the identified CNAs (n=217) were present in both primary ileal NETs and metastases. A shared CNA indicates either exactly the same CNA or overlapping CNA. B) Majority of the recurrently mutated genes (n=96) were mutated in both primary ileal NETs and metastases. C) Metastases harbored more unique recurrent noncoding variants (n=6,533) than shared with the primary ileal NETs.

Chr4p15.2 [chr4:23699756-27292161]

3.6 Mb region; 20 genes

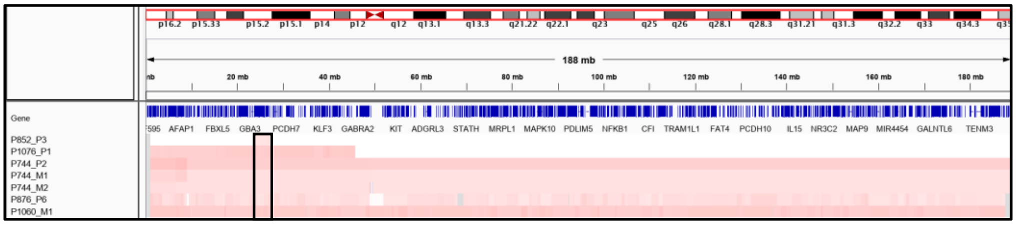

Chr10q11.21 [chr10:41911522-44544587]

2.6 Mb region; 29 genes

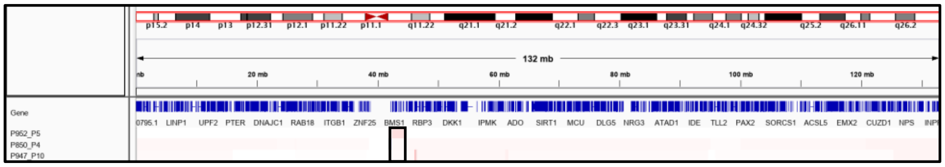

Chr14q32.2-32 [chr14:99528087-103095532]

3.6 Mb region; 80 genes

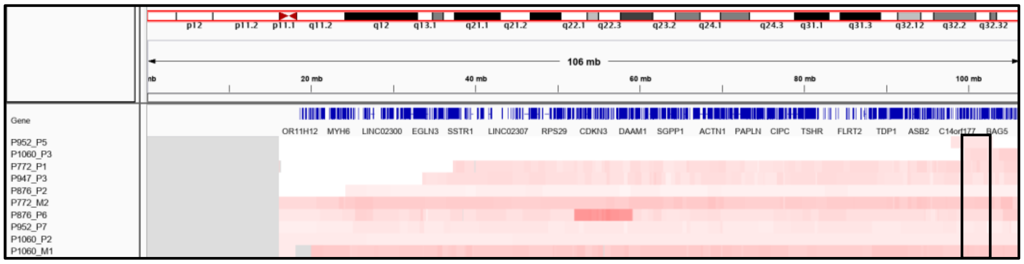

Chr18q12.2 [chr18:37090096-37238095]

0.15 Mb region; 1 gene

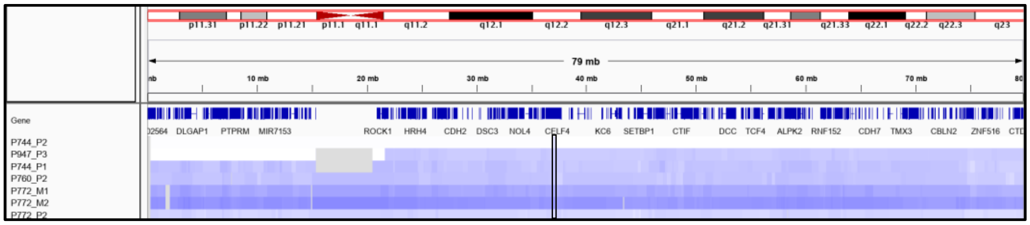

Chr19p13.11 [chr19:16820575-17850473]

1 Mb region; 34 genes

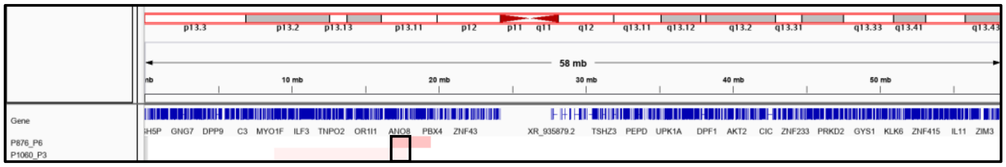

Chr20p13-12.3 [chr20:4128430-6303853]

2.2 Mb region; 25 genes

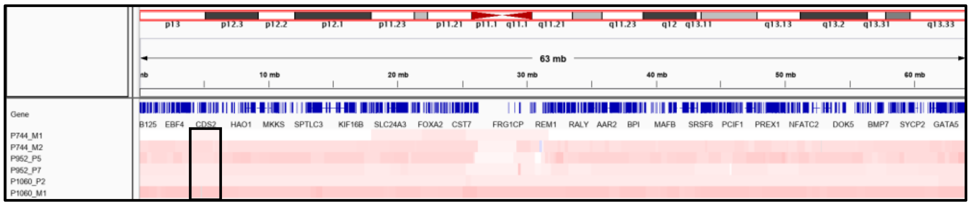

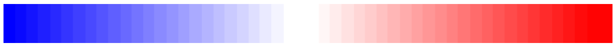

Deletion **Neutral** **Amplification**

Figure S3. Minimally targeted regions of size < 5Mb. Each minimally targeted region was observed in at least two multifocal ileal NET patients. Images of the minimally targeted regions on chr4, 14, 18 and 20 include five examples of tumors with whole chromosome alterations.

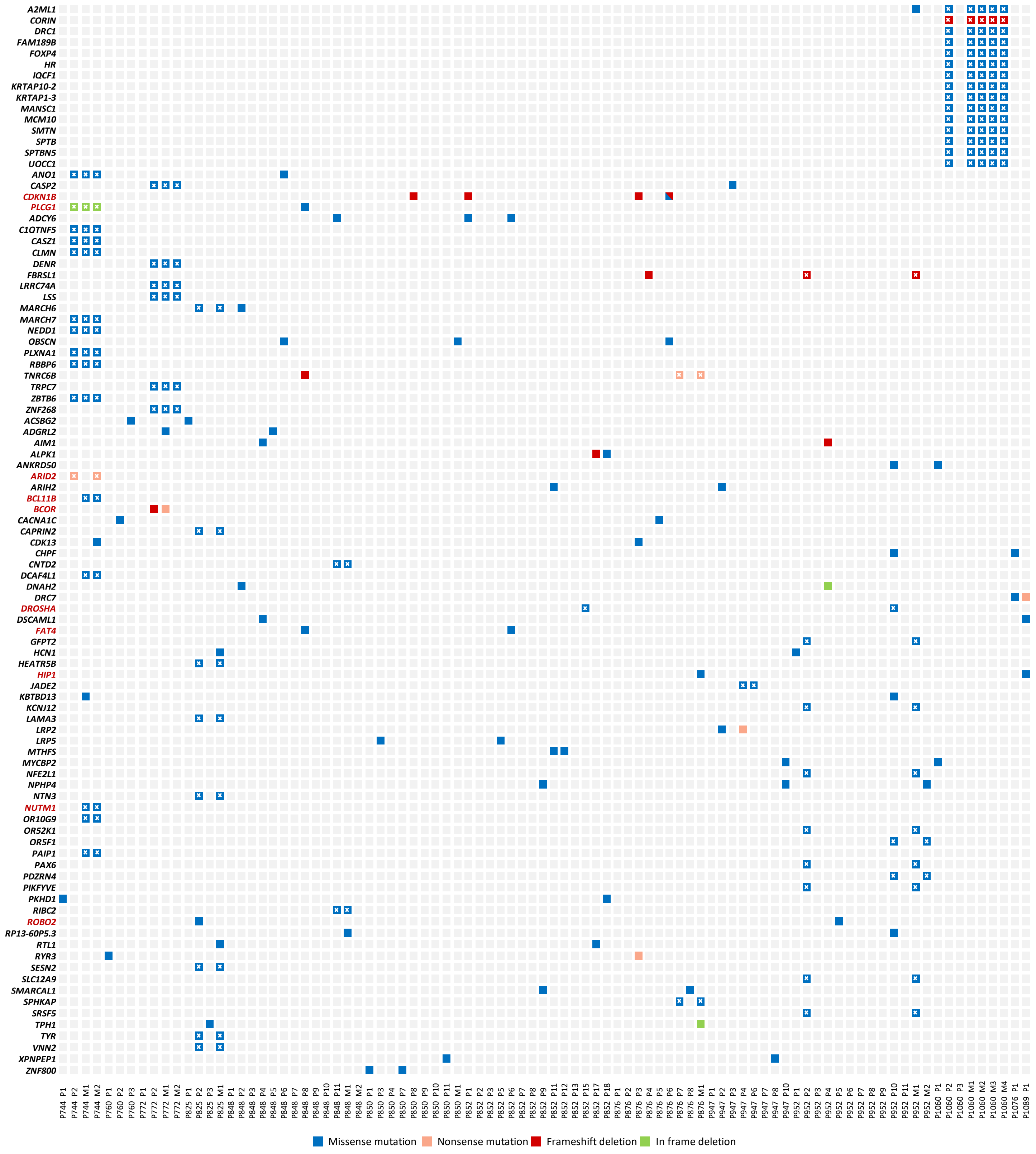

Figure S4. Recurrently mutated genes in 75 primary ileal NETs and 15 metastases. The recurrently mutated genes (n=96) have been ordered based on the frequency of the mutated tumors. Gene names marked with red are known cancer genes (COSMIC Cancer Gene Census v92) and colored boxes marked with a white cross indicate that the tumors harbor a variant in the same genomic location.

CDKN1B (chr12:12,715,058-12,721,204)

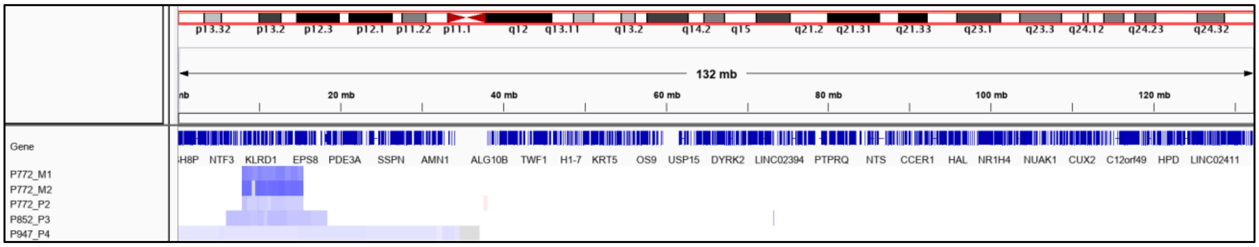

Copy-neutral LOH (P876_P6)

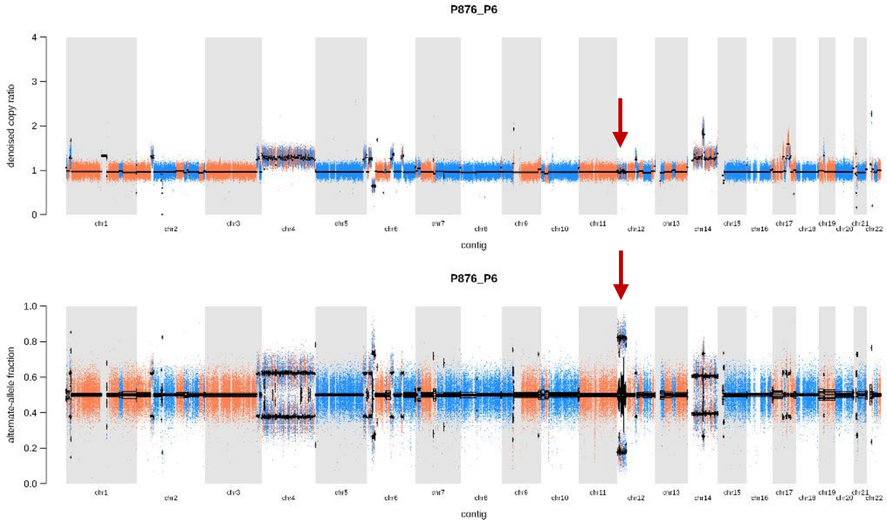

TNRC6B (chr22:40,177,925-40,335,808)

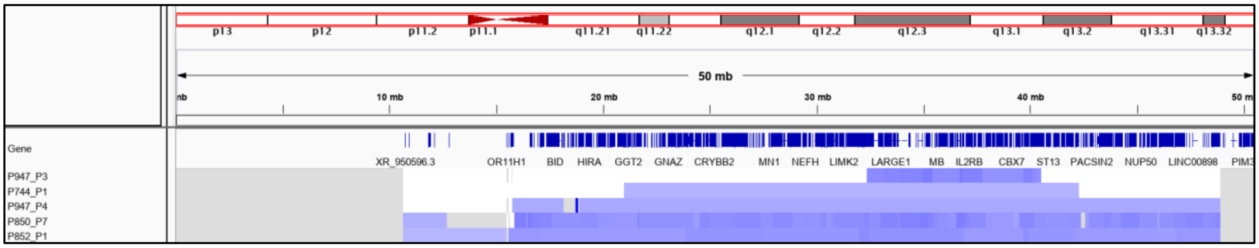

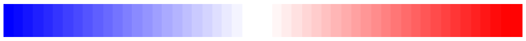

Deletion **Neutral** **Amplification**

Figure S5. Deletions affecting *CDKN1B* and *TNRC6B*. *CDKN1B* was deleted in five primary ileal NETs from three multifocal ileal NET patients. Additionally, one primary ileal NET (P876_P6) displayed copy-neutral LOH on chr12p. *TNRC6B* was affected by a deletion in five primary ileal NETs from four multifocal ileal NET patients.

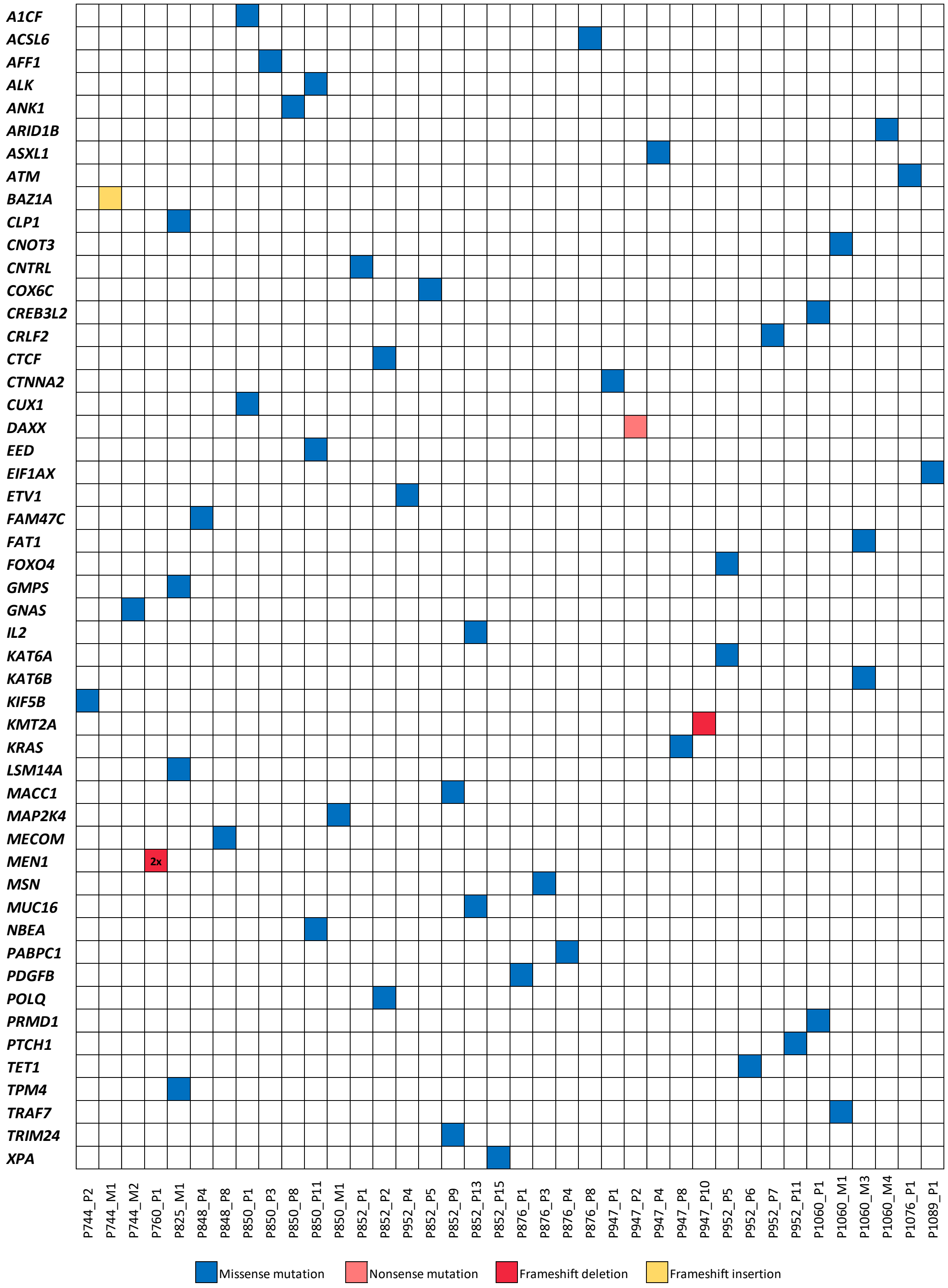

Figure S6. Known cancer genes mutated in single primary ileal NETs and metastases. Each of the known cancer genes in this table is mutated only in one tumor sample.

A.

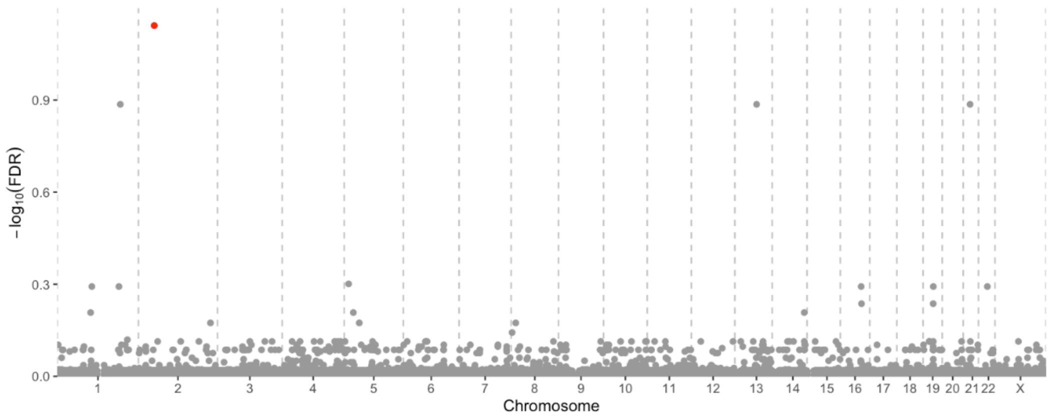

Chr	Start position	End position	Width	P-value	FDR	Count
2	48340001	48350000	10000	2.80E-07	0.072	8
21	20980001	20990000	10000	1.60E-06	0.13	7
13	66640001	66650000	10000	1.80E-06	0.13	7
1	193350001	193360000	10000	2.10E-06	0.13	7
5	13510001	13520000	10000	9.80E-06	0.5	6

B.

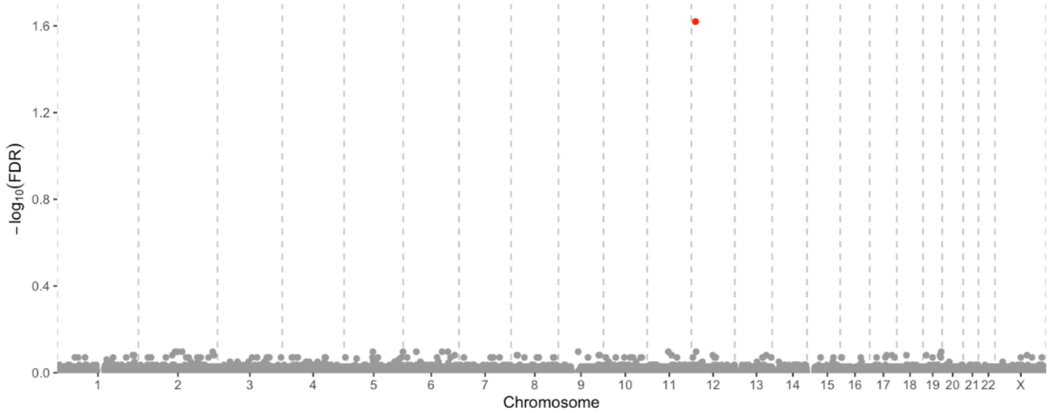

Chr	Start position	End position	Width	P-value	FDR	Count
12	12700001	12800000	100000	9.00E-07	0.024	5
6	118800001	118900000	100000	8.20E-05	0.8	3
2	227000001	227100000	100000	0.00016	0.8	3
9	60500001	60600000	100000	2.00E-04	0.8	4
6	39900001	40000000	100000	0.00022	0.8	3

Figure S7. Statistically enriched regions of SNVs and indels in 75 primary ileal NETs and 15 metastases. A) Results of a fishHook analysis for SNVs. Five top hits are reported under the scatter plot, one of them being statistically significant after multiple testing correction. B) Results of a fishhook analysis for indels. Five top hits are reported under the scatter plot, one of them being statistically significant after multiple testing correction. FDR < 0.1 was considered statistically significant.

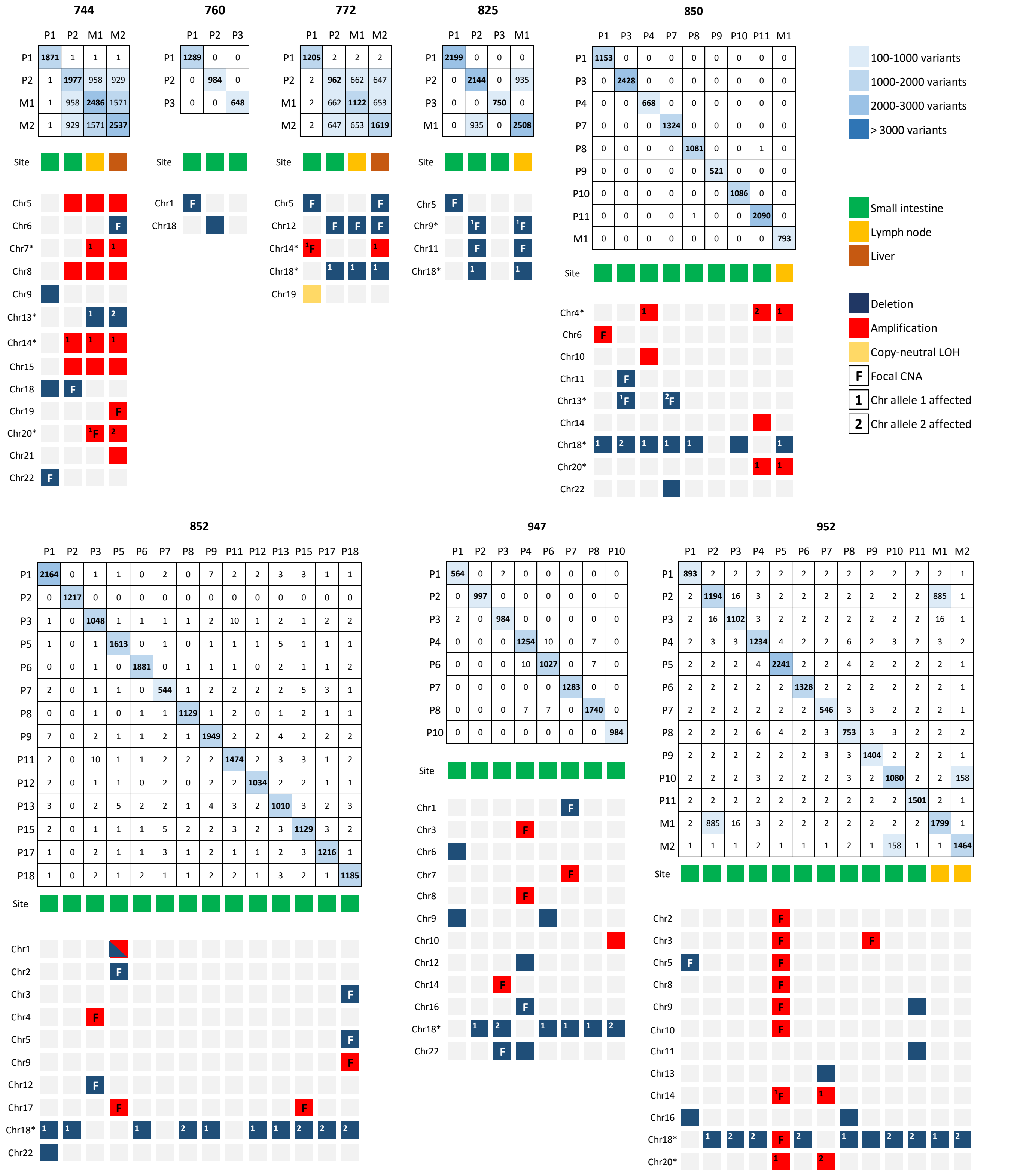

Figure S8. Somatic tumor evolution in multifocal ileal NET patients. Upper panel shows shared SNVs and indels between each tumor pair within a multifocal ileal NET patient. The somatic nature of the shared SNVs and indels was verified each time < 100 variants were shared between two tumors. Also, the presence of the shared variants in other tumors of the same patient were checked not to miss subclonal variants. Tumor sites are indicated by colored boxes. Lower panel consists of somatic CNAs identified in each tumor. The CNAs are arranged in chromosome number order. Focal CNAs are marked with 'F' to differentiate them from whole chromosome/chromosome arm events. Chromosomes marked with an asterisk contain results from the chromosome mapping analysis. P, primary tumor; M, metastasis.
